# Nitrous Oxide Causing Subacute Combined Degeneration: A Case Report

**DOI:** 10.7759/cureus.74871

**Published:** 2024-11-30

**Authors:** Nevin Thomas, Ravi Patel, Babaniji Oluwadamilola

**Affiliations:** 1 Internal Medicine, Methodist Dallas Health System, Dallas, USA

**Keywords:** b12 deficiency, n2o, scd, substance abuse, whippets

## Abstract

This case report describes a 31-year-old male who developed subacute combined degeneration as a result of vitamin B12 deficiency caused by recreational use of nitrous oxide ("whippets") over a six-month period. nitrous oxide, widely available and often used for its euphoric effects, can lead to alterations in B12 metabolism and decreased myelination, particularly in the dorsal columns, with prolonged use. Despite prompt diagnosis and treatment, including intramuscular B12 injections and physical therapy, he experienced residual weakness and required outpatient rehabilitation. This case highlights the importance of public education about the risks of nitrous oxide abuse, as its use appears to be increasing, in order to prevent further hospitalizations and morbidity.

## Introduction

Nitrous oxide (N₂O) is a biologically active compound with diverse physiological roles, including involvement in hormone signaling and smooth muscle control. This gas is commonly used for its euphoric and dissociative effects, which are attributed to its ability to block N-methyl-D-aspartate receptors [1]. N₂O is rapidly absorbed and widely utilized as an anesthetic in medical and dental settings. However, prolonged exposure to N₂O can have detrimental consequences, making its misuse a significant public health concern due to its widespread availability and ease of access [2]. Indeed, studies have reported a concerning rise in the abuse of N₂O, with prevalence estimates of 29.4% in the United States, and it is the eighth most commonly used substance in the United Kingdom [3]. N₂O can be found in readily accessible products such as whipped cream dispensers and other aerosol canisters, further contributing to the risk of misuse. Importantly, N₂O can oxidize the cobalt salts in vitamin B12, rendering this essential nutrient functionally deficient [4]. One major consequence of N₂O abuse is subacute combined degeneration (SCD) caused by vitamin B12 deficiency. SCD results from the demyelination that leads to degeneration of the dorsal and lateral columns of the spinal cord [5]. B12 is important for the integrity of myelin sheaths surrounding neurons, producing cofactors that stabilize and assist with myelin function. In this case, we discuss the neurologic deficits that may arise from SCD, including ataxia, paresthesia, weakness, and coordination deficits. Full recovery may not always be possible in this condition, depending on the chronicity of symptoms. This emphasizes the importance of prompt diagnosis and treatment to allow for complete recovery back to a patient’s baseline.

The swift onset of neurological symptoms in this patient, despite only six months of N₂O use, highlights the need for increased public awareness and education regarding the potential dangers of N₂O misuse, particularly among vulnerable populations such as younger adults.

## Case presentation

**Figure 1:**
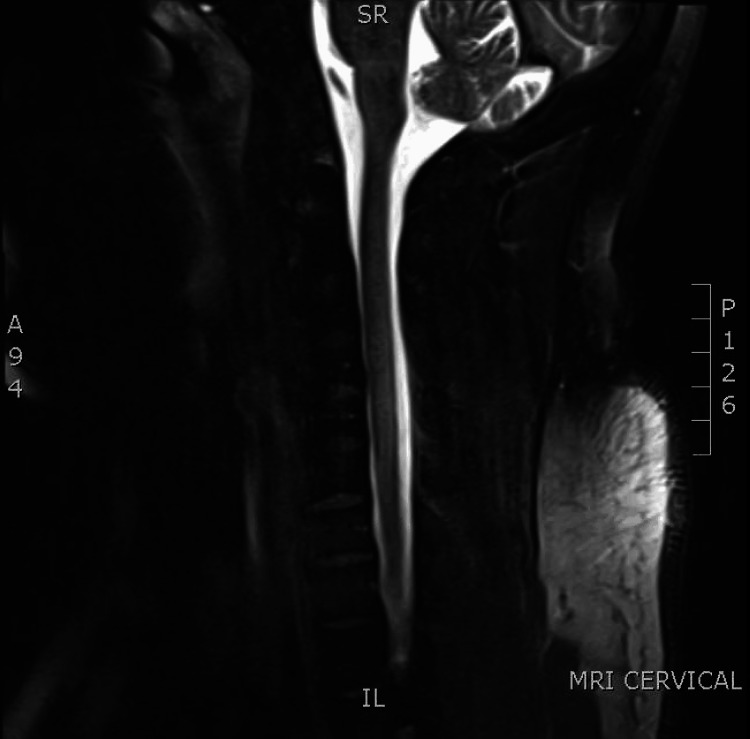
T2 hyperintense cord signal with expansion from C1 to C6 in the central and posterior cord along the dorsal columns

The case report describes a 31-year-old male patient with no significant medical history who presented with subacute onset of numbness and tingling throughout his body. The paresthesias began one month before hospital admission and progressed to include numbness from his shoulders to his toes. The patient reported that his upper and lower extremities felt unstable, and he disclosed using approximately 1 liter of N₂O for six months before the onset of symptoms. Outpatient testing revealed a vitamin B12 deficiency, with levels less than 150 pg/mL (reference range: 211-911 pg/mL). Upon admission, the patient was also found to be anemic, with a hemoglobin level of 12 g/dL (reference range: 13.5-17.5 g/dL) and a mean corpuscular volume of 98.3 fL (reference range: 80.0-100.0 fL). Folate levels were elevated at greater than 20 ng/mL (reference range: 3.0-20.0 ng/mL), while the comprehensive metabolic panel was unremarkable. Magnetic resonance imaging of the spine, performed with and without contrast, revealed abnormal signals in the dorsal spinal cord extending from the C1-C2 to C6-C7 levels, findings consistent with subacute combined degeneration (Figure 1). 

Neurology was consulted during the admission, and the patient was diagnosed with subacute combined degeneration secondary to N₂O abuse, supported by imaging findings and clinical presentation. Treatment included intramuscular vitamin B12 injections, starting with 1000 mcg daily for seven days, followed by 1000 mcg weekly for four weeks, before transitioning to oral vitamin B12 supplementation. Physical therapy was involved throughout the hospitalization and identified significant coordination deficits in all extremities. Post-hospital rehabilitation was recommended due to the patient’s good rehabilitation potential. Despite the interdisciplinary approach and prompt diagnosis and treatment, outpatient rehabilitation was ultimately declined because of insurance approval issues. However, the patient was cleared by physical therapy for discharge with outpatient therapy. He was counseled extensively on the importance of abstaining from further N₂O misuse.

## Discussion

Nitrous oxide (N₂O), commonly known as "whippets," is a legal substance that is increasingly being abused due to its euphoric effects, which can be easily obtained from stores or online. This growing trend has become a significant public health concern, particularly for vulnerable populations, necessitating both social and legislative action. This case report presents the deleterious and long-term consequences of prolonged use of this substance. The most common symptoms associated with this condition include instability in walking, limb numbness, and ataxia [6]. The underlying mechanism involves the oxidation of cobalt in vitamin B12 or cobalamin by N₂O, which irreversibly inactivates the vitamin [4]. This, in turn, leads to an increase in methylmalonic acid, resulting in decreased myelination, particularly in the dorsal columns. This vitamin B12 deficiency, even after a relatively short period of sustained N₂O use, can lead to glial cell dysfunction and demyelination, ultimately resulting in the development of subacute combined degeneration of the central nervous system, as demonstrated in the present case report [7]. This is often characterized by neuroimaging, which may reveal striped and speckled lesions, while cerebrospinal fluid findings are typically normal [6].

Common neurological manifestations of vitamin B12 deficiency include sensorimotor polyneuropathy, ataxia, and psychosis. The clinical presentation can less commonly present as a myelopathy affecting the cervical spine, akin to the subacute combined degeneration syndrome associated with pernicious anemia. The patient's clinical presentation, encompassing neurological symptoms such as paresthesias and ataxia, as well as the corresponding imaging and laboratory findings, was consistent with this neurological condition. With vitamin B12 supplementation and physical therapy, the patient gradually improved during hospitalization. This case emphasizes that prompt diagnosis and initiation of treatment from the outset can lead to rapid improvements in clinical symptoms. Interdisciplinary collaboration, including physical therapy and neurology, facilitated a higher level of effective care. Vitamin B12 deficiency should be considered in the differential diagnosis for patients presenting with similar symptoms, as it represents a reversible disease process. The recommended treatment for the neurological dysfunction resulting from vitamin B12 deficiency is typically parenteral supplementation. A suggested dosing regimen involves an initial loading phase of 1000 mcg of vitamin B12 every other day for two weeks, followed by a transition to oral and parenteral supplementation [5]. Symptoms can be reversed if N₂O abuse is only ongoing for a few months, but prolonged use and subacute combined degeneration may lead to lifelong disability [8]. It is crucial for public health, especially among adolescents, to abstain from N₂O use, as it may result in irreparable long-term effects. Preventing the use of this substance may decrease individual morbidity outcomes and reduce the healthcare burden in the future.

## Conclusions

This case underscores the need for public education regarding the risks associated with the use of nitrous oxide, a substance often perceived as harmless and readily available. Prolonged abuse of nitrous oxide, colloquially known as "whippets," can lead to serious and potentially irreversible consequences, such as vitamin B12 deficiency and the development of subacute combined degeneration. Raising awareness about the detrimental effects of nitrous oxide misuse is crucial to mitigating further hospitalizations and morbidity related to its use.
